# HDACi mediate UNG2 depletion, dysregulated genomic uracil and altered expression of oncoproteins and tumor suppressors in B- and T-cell lines

**DOI:** 10.1186/s12967-020-02318-8

**Published:** 2020-04-07

**Authors:** Tobias S. Iveland, Lars Hagen, Animesh Sharma, Mirta M. L. Sousa, Antonio Sarno, Kristian Lied Wollen, Nina Beate Liabakk, Geir Slupphaug

**Affiliations:** 1 Department of Clinical and Molecular Medicine, Faculty of Medicine and Health, Norwegian University of Science and Technology, 7491 Trondheim, Norway; 2 Cancer Clinic, St. Olav’s Hospital, Trondheim, Norway; 3 Clinic of Laboratory Medicine, St. Olav’s Hospital, Trondheim, Norway; 4 Proteomics and Modomics Experimental Core, PROMEC, at NTNU and the Central Norway Regional Health Authority, Stjørdal, Norway

**Keywords:** Histone deacetylase inhibitors, HDACi, Lymphoma, PCLAF, SILAC, Uracil-DNA glycosylase, UNG2, Pyrimidine metabolism, HIV-1

## Abstract

**Background:**

HDAC inhibitors (HDACi) belong to a new group of chemotherapeutics that are increasingly used in the treatment of lymphocyte-derived malignancies, but their mechanisms of action remain poorly understood. Here we aimed to identify novel protein targets of HDACi in B- and T-lymphoma cell lines and to verify selected candidates across several mammalian cell lines.

**Methods:**

Jurkat T- and SUDHL5 B-lymphocytes were treated with the HDACi SAHA (vorinostat) prior to SILAC-based quantitative proteome analysis. Selected differentially expressed proteins were verified by targeted mass spectrometry, RT-PCR and western analysis in multiple mammalian cell lines. Genomic uracil was quantified by LC–MS/MS, cell cycle distribution analyzed by flow cytometry and class switch recombination monitored by FACS in murine CH12F3 cells.

**Results:**

SAHA treatment resulted in differential expression of 125 and 89 proteins in Jurkat and SUDHL5, respectively, of which 19 were commonly affected. Among these were several oncoproteins and tumor suppressors previously not reported to be affected by HDACi. Several key enzymes determining the cellular dUTP/dTTP ratio were downregulated and in both cell lines we found robust depletion of UNG2, the major glycosylase in genomic uracil sanitation. UNG2 depletion was accompanied by hyperacetylation and mediated by increased proteasomal degradation independent of cell cycle stage. UNG2 degradation appeared to be ubiquitous and was observed across several mammalian cell lines of different origin and with several HDACis. Loss of UNG2 was accompanied by 30–40% increase in genomic uracil in freely cycling HEK cells and reduced immunoglobulin class-switch recombination in murine CH12F3 cells.

**Conclusion:**

We describe several oncoproteins and tumor suppressors previously not reported to be affected by HDACi in previous transcriptome analyses, underscoring the importance of proteome analysis to identify cellular effectors of HDACi treatment. The apparently ubiquitous depletion of UNG2 and PCLAF establishes DNA base excision repair and translesion synthesis as novel pathways affected by HDACi treatment. Dysregulated genomic uracil homeostasis may aid interpretation of HDACi effects in cancer cells and further advance studies on this class of inhibitors in the treatment of APOBEC-expressing tumors, autoimmune disease and HIV-1.

## Background

Lysine acetylation is catalyzed by histone acetyltransferases (HATs) and reversed by histone deacetylases (HDACs). Despite their names, these enzymes also target a wide range of non-histones and more than 6000 human proteins are currently known to be acetylated ([1], http://plmd.biocuckoo.org/). Dysregulated activity of HATs and HDACs have been associated with multiple cancers and often associated with poor prognosis [2]. Thus, inhibitors of these enzymes (HATi and HDACi, respectively) have become attractive candidates in cancer treatment. The four FDA-approved anti-cancer HDACi vorinostat (SAHA), panobinostat, romidepsin and belinostat are, as of this time, restricted to cutaneous T cell lymphoma (CTCL), and multiple myeloma (a plasma cell malignancy). In addition, entinostat (MS-275) is entering phase III trial in patients with hormone receptor positive advanced breast cancer [3] and tucidinostat has showed increased progression free survival in the same patient group [4]. Within other diseases, the pan-HDACi valproate has been used for decades in epilepsy treatment and HDACi has been suggested as a potential cure for HIV by purging latent reservoirs [5], and a treatment of B-cell driven autoimmunity [6].

Despite the success of HDACi in treatment of the above B- and T-lymphocyte-derived malignancies, mixed results have been observed in clinical trials involving other cancers. The molecular mechanisms underlying this remain elusive and likely involve cell- and tissue variability in baseline HDAC activities as well as the preceding chromatin state. HDACi have been shown to affect several biological processes associated with malignancy such as DNA repair, cell differentiation and cell death [7, 8] and that may vary between cancers. Most of these studies have focused on transcriptome alterations, which yield limited information on protein levels. Given the potential of lysine acetylation to modulate protein turnover by masking of ubiquitinylation targets, surprisingly few studies have monitored proteome-wide alterations after HDACi treatment. To the best of our knowledge, only one previous study has profiled proteome alterations subsequent to HDACi treatment of lymphocyte-derived malignancies [9]. They identified 72 differentially expressed proteins (DEPs) after SAHA treatment of the Jurkat T-cell line, which led to induction of autophagy [9].

Here we report an unbiased comparative study of one T-leukemia (Jurkat) and one B-cell lymphoma (SUDHL5) cell line exposed to the pan-HDAC inhibitor SAHA, by employing stable isotope labeling with amino acids in cell culture (SILAC)-based quantitative proteome profiling. SILAC is often considered a “gold standard” in quantitative proteomics since labeling and sample mixing are performed at the earliest possible steps, thus eliminating potential bias from downstream sample handling. In humans this method is currently amenable to cells or cell lines that can be kept in culture until satisfactory labeling is achieved. We find 125 and 89 DEPs in Jurkat and SUDHL5, respectively, including several oncoproteins and tumor suppressors previously not reported to be affected by HDACi. Whereas phagosome maturation was reported as the top canonical pathway affected in Jurkat, in agreement with the previous study [9], pyrimidine deoxyribonucleotides de novo biosynthesis was the top affected pathway in SUDHL5. Here, altered expression of key enzymes could potentially mediate increased dUTP/dTTP ratio and increased genomic uracilation. Most notably, we observed robust downregulation of the major uracil-DNA glycosylase, UNG2. This was recapitulated with several HDACi and across human cancer cell lines of varying origin as well as in murine CH12F3 cells. In freely cycling HEK293 cells, HDACi mediated proteasomal degradation of hyperacetylated UNG2 and concomitantly increased genomic uracil. In CH12F3 cells, HDACi mediated reduced class-switch recombination (CSR), in agreement with the role of UNG2 in antibody affinity maturation. Our findings establish UNG2 as a novel and ubiquitous target for HDACi-mediated degradation. This may expand indications for HDACi towards synthetic lethality in APOBEC3B expressing tumors [10] and to increase efficiency of deaminase-mediated CRISPR-Cas9 gene therapy [11]. Our results may also aid efforts to purge HIV viruses from latent reservoirs and treatment of autoimmune disease.

## Materials and methods

### Antibodies and reagents

Primary antibodies: Anti-UNG: Polyclonal PU059 (in-house, 0.5 µg/ml), monoclonal 2C12 (OriGene, Rockville, MD, TA503563, 0.5 µg/ml) and 1G2 (OriGene TA503755, 0.5 µg/ml) (all recognizing catalytic domain of UNG1/2), in-house polyclonal mouse anti-murine UNG. Monoclonal anti-β-actin (Abcam, Cambridge, UK, Ab8226, 0.5 µg/ml), monoclonal anti-PCNA (Santa Cruz Biotechnology, Santa Cruz, CA, sc-56, 0.2 µg/ml), polyclonal anti-p21 (Abcam Ab18209, 0.2 µg/ml), polyclonal anti-acetyllysine (Abcam Ab21623, 0.25 µg/ml). Secondary antibodies according to the manufacturer’s recommendation: Swine anti-rabbit, HRP (Dako, Glostrup, Denmark), rabbit anti-mouse, HRP (Dako) or IRDye 680RD/800CW anti-mouse/rabbit (Li-Cor). Vorinostat (SAHA) (Cayman Chemical, Ann Arbor, MI), 10009929), Entinostat (MS-275) (Selleck Chemicals, Houston, TX), S1053), valproic acid (Sigma-Aldrich, P4543). MG132 (Sigma-Aldrich, c2211), Epoxomicin (Santa Cruz, sc-201298), Bortezomib (Santa Cruz, sc-217785), Nocodazole (Sigma-Aldrich, M1404).

### Cell culture and preparation of extracts

All cell culture reagents were from Sigma‐Aldrich, St. Louis, MO, USA, unless otherwise stated. HEK293 and HeLa cells were cultured in Dulbecco’s modified Eagles medium with 10% FBS, 2 mM l-glutamine, 2.5 µg/ml amphotericin B and 0.1 mg/ml gentamicin. Jurkat and SUDHL5 cell lines were cultured in RPMI 1640 medium with 10% heat-inactivated FBS (56 °C, 30 min), 2 mM l-glutamine, 2.5 µg/ml amphotericin B and 1% PenStrep (Thermo Fisher, Waltham, MA, USA). CH12F3 cells (mouse B lymphocytes, class switch-proficient) were cultured in RPMI-1640 supplemented with 10% heat-inactivated FBS, 50 μM 2-mercaptoethanol (Gibco) and 1% PenStrep (Thermo Fisher). Where indicated, FBS was dialyzed through a 6-8 kDa membrane overnight against 1 × PBS. Jurkat and SUDHL5 cells for SILAC analysis were cultured and processed for LC–MS/MS as described separately.

For preparation of total cell extract (TCE), trypsinized cells were washed, pelleted and resuspended in 2 × packed cell volume of lysis buffer (10 mM HEPES pH 7.9, 200 mM KCl, phosphatase inhibitor cocktail 2 and 3, 1 mM EDTA, 0.5% NP-40, 0.5% Triton X-100, 2 µM MG132 and 10 µM SAHA from Sigma-Aldrich and Complete^®^ protease inhibitor from Roche, Basel, Switzerland) and subjected to horizontal rolling for 1 h at 4 °C. Lysed cells were centrifuged for 10 min at 16,000×*g* and supernatant collected as TCE. Protein was quantified by the Bradford assay (Bio-Rad) against bovine serum albumin.

### SILAC LC–MS/MS analysis

SUDHL5 and Jurkat cell lines were grown in SILAC-RPMI 1640 medium with 10% heat inactivated and dialyzed FBS (Thermo Fisher), 2 mM l-glutamine, 2.5 µg/ml amphotericin B, 1% PenStrep, as either LIGHT (l-lysine-^12^C_6_ and l-arginine-^12^C_6_) or HEAVY (l-lysine-^13^C_6_,^15^N_2_ and l-arginine-^13^C_6_,^15^N_4_) and underwent six doublings before incorporation efficiency was evaluated by mass spectrometry. Both cell lines grew well in the SILAC medium and reached > 95% incorporation of heavy amino acids prior to initiation of the experiment. Cells were lysed in 10 mM Tris–HCl pH 8, 4% SDS, 0.1 M DTT by sonication for 30 s using Branson Sonifier 450 (Branson, St. Louis, MO) with output control 2.5 and duty cycle 20%. Cell debris was pelleted by centrifugation at 13,200×*g* for 10 min and the supernatant harvested as protein extract. Protein concentration was measured using the MilliPore Direct Detect IR spectrometer. 50 µg (protein) each of HEAVY and LIGHT extract was mixed and proteins precipitated using chloroform/methanol [12]. The protein pellet was dissolved in 150 μl 50 mM NH_4_HCO_3_, reduced with 10 mM DTT for 30 min at 55 °C and further alkylated using 20 mM iodoacetamide for 30 min at room temperature in the dark. Proteins were digested using 1.5 µg trypsin (Promega Corporation, Madison, WI) at 37 °C overnight. Peptides were desalted using homemade C18 Stagetips [13].

Peptides were analyzed on a LC–MS/MS platform consisting of an Easy-nLC 1000 UHPLC system in-line with a QExactive orbitrap (Thermo Fisher) in data dependent acquisition (DDA) mode using the following parameters: electrospray voltage 1.9 kV, HCD fragmentation with normalized collision energy 30, automatic gain control (AGC) target value of 3E6 for Orbitrap MS and 1E5 for MS/MS scans. Each MS scan (m/z 400–1600) was acquired at a resolution of 70,000 FWHM, followed by 10 MS/MS scans triggered for intensities above 1.4E4, at a maximum ion injection time of 100 ms for MS and 60 ms for MS/MS scans. Peptides were injected onto a C-18 trap column (Acclaim PepMap100 (75 μm i. d. × 2 cm, C18, 3 μm, 100 Å, Thermo Fisher) and further separated on a C-18 analytical column (Acclaim PepMap100 (75 μm i. d. × 50 cm, C18, 2 μm, 100 Å, Thermo Fisher) using a gradient from 0.1% formic acid to 40% CH_3_CN, 0.1% formic acid at 250 nl/min.

### Bioinformatic analysis of SILAC MS data

Preview 2.3.5 (Protein Metrics Inc. https://www.proteinmetrics.com) was used to determine optimal search criteria. These were plugged in MaxQuant [14] v 1.5.7.4 with SILAC multiplicity of 2 (Arginine 10 and Lysine 8) mapping the spectra over Human canonical proteome including isoforms [15] (Jan 2017 update). The following search parameters were used: enzyme specified as trypsin with maximum of 2 missed cleavages allowed; Deamidation of Asp/Glu, oxidation of Met and N-terminal acetylation as variable- and carbamidomethylation of Cys as a fixed modification. Mass tolerance was set to 20 ppm with False Discovery Rate < 0.01 (high confidence) for PSM, peptide as well as protein group identification. The normalized SILAC ratios of identified protein groups were log transformed with base 2 and replicates were collapsed to a median representative value. Student’s t-test was conducted over these values and relative up/down-regulated proteins (absolute fold-change > 1.5 and p-value < 0.05) were presented to Ingenuity Pathways Analysis tool (Ingenuity^®^ Systems, https://www.qiagenbioinformatics.com/products/ingenuity-pathway-analysis/) to find significantly affected pathways.

The mass spectrometry proteomics data have been deposited to the ProteomeXchange Consortium [16] via the PRIDE partner repository with the dataset identifier PXD00008293.

### Targeted PRM analysis

For targeted PRM analysis, 50 µg protein was incubated with 5 mM Tris (2-carboxyethyl) phosphine (TCEP) for 30 min and alkylated with 1 µmol/mg protein of iodoacetamide for 30 min in the dark. Proteins were precipitated using chloroform/methanol [17], repeatedly reduced/alkylated and digested overnight with trypsin (Promega) at 1:50 ratio (w/w, enzyme:protein) at 37 °C. Tryptic digests were dried, resuspended in 0.1% formic acid and analyzed on the Easy-nLC 1000 QExactive mass spectrometer system operating in targeted-MS2 mode. Peptides were separated using a gradient from 0.1% formic acid to 40% CH_3_CN, 0.1% formic acid at 250 nl/min over 100 min. The QExactive was operating in positive-ion mode using electrospray voltage 1.9 kV and HCD fragmentation. Each MS/MS scan was acquired at 35,000 FWHM, normalized collision energy (NCE) 28, automatic gain control (AGC) target value of 2E5, maximum injection time of 120 ms and isolation window 2 m/z. All PRM methods were designed, analyzed, and processed using Skyline software version 3.1.0.7382 [18]. In silico selection of proteotypic peptides was performed via Skyline using the *Homo sapiens* reference proteome (www.uniprot.org) to exclude non-unique peptides. Synthetic purified peptides (Thermo Fisher) and tryptic digests from recombinant proteins were analyzed as above. Information on retention time and fragmentation of the top 2–6 ionizing tryptic peptides (2 + or 3 + charge states) for each protein were used to build a scheduled method with a retention time window of 4 min. The sum of the integrated peak areas of the 4 most intense fragments was used for peptide quantification. Peptide areas for multiple peptides of the same protein were summed to assign relative abundance. The given error bars represent the standard deviation of 3 biological replicates and p-values were calculated using non-paired student t-test.

### Quantification of genomic uracil

Quantification of genomic uracil was performed essentially as described [19]. Briefly, DNA was isolated by phenol:chloroform:isoamyl extraction, treated with alkaline phosphatase to remove free deoxyribonucleosides, and then enzymatically hydrolyzed to deoxyribonucleosides. Deoxyuridine was separated from deoxycytidine by HPLC fractionation using a reverse-phase column with embedded weak acidic ion-pairing groups (2.1 mm × 150 mm, 5 μm, Primesep 200, SIELC Technologies, Wheeling, IL), using a water/acetonitrile gradient containing 0.1% formic acid. The dU fraction was finally analyzed by ESI-LC/MS/MS using a reverse phase column (2.1 mm × 150 mm, 3.5 μm, Zorbax SB-C18, Agilent Technologies, Santa Clara, CA), using a water/methanol gradient containing 0.1% formic acid on an API5000 triple quadrupole mass spectrometer (Applied Biosystems) in positive ionization mode. UNG-treated DNA (background control) was prepared as described [20]. Experiments were performed as biological triplicates with standard deviation representing the error bars and p-value calculated with non-paired t-test.

### Cell cycle analysis and live cell sorting

For cell cycle sorting, live cells were stained with Vybrant Violet DyeCycle Stain according to the manufacturers protocol (Thermo Fisher). Cells in different cell cycles phases (G1, S and G2) were sorted in a BD FACS Aria II cell sorter (BD Biosciences, San Jose, CA) using the violet laser (405 nm) for excitation, the pacific blue channel (BP 450/40 nm) for emission and a 100 µm nozzle at a rate of 1500 events/second. Regular cell cycle analysis was performed by standard procedure with RNase A and propidium iodide (Thermo Fisher) and analyzed in a BD FACS Canto (BD Biosciences).

## 2D and 1D polyacrylamide gel electrophoresis, western analysis and immuno-confocal microscopy

TCEs were diluted in DeStreak (GE Healthcare, Chicago, IL) and cup-loaded to Immobiline DryStrips (7 cm, pH 7-11) (GE Healthcare) according to the manufacturer’s instructions, followed by LDS-PAGE in the second dimension. For 1D LDS-PAGE, proteins were electrophoresed in 10% NUPage Novex Bis–Tris gels (Life Technologies, Carlsbad, CA) with MOPS running buffer. Gels were electroblotted to Immobilon PVDF membranes (Millipore, Burlington, MA). For immunostaining (room temperature), membranes were blocked for 1 h in PBST with 5% fat-free dry milk and incubated with primary antibody in blocking buffer for 1 h. After 3 × 10 min washing in PBST, membranes were incubated for 1 h in secondary antibody in blocking buffer. For HRP-conjugated secondary antibodies, membranes were washed 3 × 10 min in PBST, developed using SuperSignal West Femto (Thermo Fisher) and visualized using Kodak Image station 4000R. For IRDye secondary antibodies, imaging was performed on a LI_COR Odyssey imager (LI-COR, Lincoln, NE) after 1 h of incubation, 2 × PBST wash and 2 × PBS washes. For immuno-confocal analysis, adherent cells were grown in plates with #1.5 cover glass (Mattek p35g-1.5-14-c), fixed in 2% paraformaldehyde for 10 min and incubated in methanol (− 20 °C) for 20 min. Incubation and washing steps were performed in blocking buffer (PBS, 2% FBS) unless otherwise stated. Cells were washed/blocked for 3 × 5 min at room temperature, incubated with 5 µg/ml PU59 antibody at 37 °C, 60 min, washed 3 × 5 min and incubated with alexa fluor 647 goat anti-rabbit antibody (Thermo Fisher, 1:2000) at 37 °C for 45 min. After 3 × 10 min washing, cells were covered with 1 µg/ml DAPI (Thermo Fisher) in PBS for 30 min at room temperature and washed 3 × 10 min in PBS. Confocal images were acquired on a Leica TCS SP8 system attached to a DMI 6000 inverted microscope, equipped with a HC Pl APO CS2 63x/1.40 oil objective (Leica Microsystems, Mannheim, Germany).

### RT-PCR

RNA was isolated using mirVana RNA isolation kit (Ambion, Austin, TX) according to manufacturer’s protocol. RNA concentration and quality were measured on a NanoDrop ND-1000 spectrophotometer (Thermo Fisher). Total RNA (770 ng) was reverse transcribed using TaqMan according to the manufacturer’s instructions (Applied Biosystems, Beverly, MA). UNG2 mRNA was quantified using UNG2 specific primers (Hs01037094_m1) and qPCR carried out on a Chromo4 (Bio-Rad, Hercules, CA) real-time PCR system. Relative expression was calculated by the ΔCt method using GAPDH as endogenous control.

### Class switch recombination assay

Briefly, approximately 2000 CH12F3 cells (10,000 cells/ml) were seeded in flat-bottomed 96-well plates in 200 µl growth medium. Cells were stimulated with 2 μg/ml hamster anti mouse CD40 (BD Biosciences), and 10 ng/ml murine recombinant IL-4 (Peprotech, Rocky Hill, NJ), and 1 ng/ml human recombinant TGF-β1 (Peprotech) for 4 days. The cells were then stained with LIVE/DEAD red viability stain (Thermo Fisher), blocked with Fc receptor antibody (2.4G2) (Thermo Fisher), fixed and permeabilized in CytoFix/Cytoperm (BD Biosciences), and washed in PermWash (BD Biosciences) containing saponin. Anti-mouse-IgA-PE (Thermo Fisher eBioscience, clone 11-44-2) was used for cytoplasmic staining and detection of IgA. Cells were washed with PermWash and suspended in 200 μl of PBS before analysis. Samples, unstained and single stained controls, were analyzed on a BD FACS Canto (BD Biosciences). Viable CH12F3 cells were analyzed for IgA expression using FlowJo^®^ version 7.6 for PC software.

## Results

### HDAC inhibition affects a common subset of oncoproteins and tumor suppressors in B- and T-cell lymphoma cell lines

**Fig. 1:**
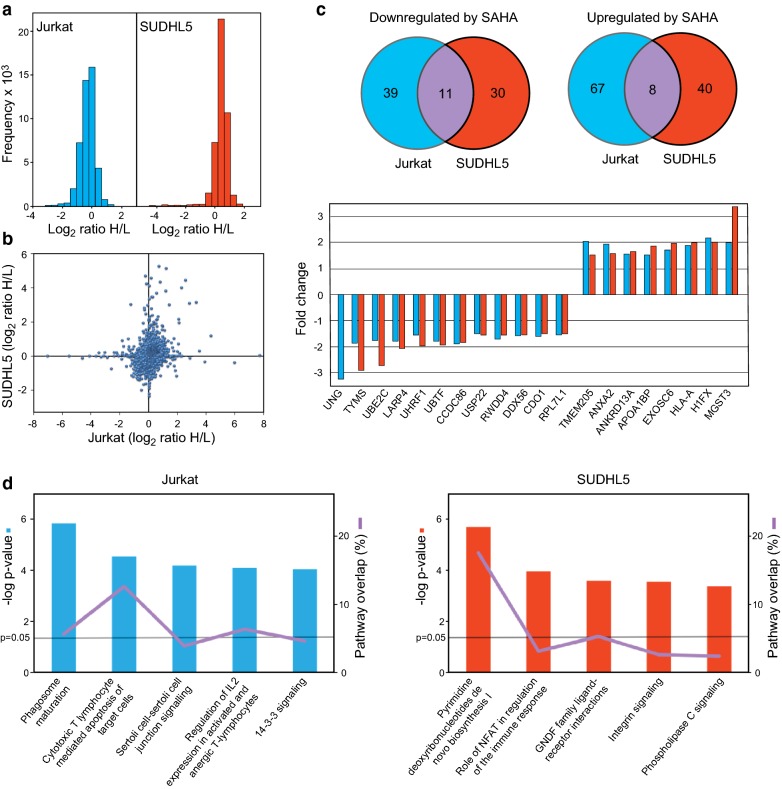
**a** Raw distribution of SILAC quantifications given as log_2_ (heavy (control)/light (SAHA) ratios. **b** Scatterplot of the normalized SILAC data showing protein expression changes (log_2_ scale) in Jurkat (x-axis) and SUDHL5 (y-axis) after SAHA treatment. **c** Venn diagrams illustrating significant differentially expressed proteins in Jurkat and SUDHL5 after SAHA treatment (upper panel). The lower panel shows the 19 common proteins that were significant differentially (> 1.5-fold) expressed in the two cell lines (linear scale). **d** DEPs from Jurkat (left) and SUDHL5 (right) were presented to IPA analysis. The most confidently affected pathways are shown with -log p values on the Y-axis, indicated with a line is p = 0.05. The percentage of overlap of the pathway is also denoted

Jurkat and SUHDL5 cells were treated with 2 µM SAHA or DMSO vehicle (Ctrl.) for 24 h and peptides quantified by Orbitrap QExactive mass spectrometry (MS). MaxQuant [14] analysis identified 4646 and 4349 proteins in Jurkat and SUDHL5, respectively, of which 3913 and 3190 proteins were quantified (PEP < 0.01) (Additional file 1: Table S1). The non-normalized log_2_ distributions are shown in Fig. 1a and a scatterplot of the SILAC data illustrated in Fig. 1b. SAHA treatment resulted in 125 and 89 differentially expressed proteins (DEPs) in Jurkat and SUDHL5 respectively, and 19 common to both cell lines (Fig. 1c). The common DEPs are also listed in Table 1 with their potential relevance to lymphomagenesis and/or response to HDACi treatment.

**Table 1:** DEPs common to Jurkat and SUDHL5 after SAHA treatment, and their potential relevance to lymphomagenesis and/or response to HDACi treatment (fold changes are given in Fig. 1c)

Gene symbol	Protein name	Potential relevance to lymphomagenesis and/or HDACi treatment
*Common upregulated*		
MGST3	Microsomal glutathione S-transferase 3	Oxidant detoxification. Potentially upregulated as a response to HDAC-induced activation of Nrf2
H1FX	Histone 1 FX	Elusive
HLA-A	HLA class I histocompatibility antigen, A-69 alpha chain	Several HLA class I alleles and variants have been associated with lymphoma development (reviewed in [86])
EXOSC6	Exosome complex component MTR3	The RNA exosome has been implicated in both Ig CSR and SHM by targeting AID to transcribed dsDNA substrates and thus regulating its mutator activity [87]
NAXE	NAD(P)H-hydrate epimerase	NAXE (AI-BP) binds APOA1 and inhibits VEGFR2 signaling by promoting cholesterol efflux from caveolae/lipid rafts [88]
ANKRD13A	Ankyrin repeat domain-containing protein 13A	Part of the BCR signalosome. Promotes endocytosis of EGFR [89]
ANXA2	Annexin A2	ANXA2 promotes binding of lymphoma cells to epithelial cells [90]
TMEM205	Transmembrane protein 205	Increases resistance to cisplatin, potentially by exocytosis of platinum-containing vesicles [91]
*Common downregulated*		
UNG*	Uracil-DNA glycosylase	Major DNA glycosylase for excision of genomic uracil. Deficiency associated with increased lymphoma risk in mice [92]
TYMS	Thymidylate synthase	Essential in *de novo* dTTP synthesis. Target of cancer antimetabolites. Decreased levels improves response to 5-fluorouracil [93]
UBE2C	Ubiquitin-conjugating enzyme E2 C	Correlated to tumor aggressiveness in a variety of lymphomas and its downregulation may contribute to the clinical efficacy of HDAC inhibitors in such malignancies [94]
LARP4	La-related protein 4	Tumor suppressor that may reduce metastatic potential [95]
UHRF1	E3 ubiquitin-protein ligase UHRF1	Reader of DNA and histone methylation that is essential to proliferation and is overexpressed in many cancers [96]
UBTF	Nucleolar transcription factor 1	Transcription factor for ribosomal RNA. Depletion induces DNA damage and genomic instability [97]
CCDC86	Coiled-coil domain-containing protein 86	Cooperates with Myc to drive aggressive lymphoma growth. Knockdown sensitizes lymphoma B cells to Rituximab [98]
USP22	Ubiquitin carboxyl-terminal hydrolase 22	Histone deubiquitinase and component of the SAGA HAT complex [99]. Putative stem-cell marker in ALL [100]
RWDD4	RWD domain-containing protein 4	Downregulation suppresses proliferation and invasion in bladder cancer cells [101]
DDX56	Probable ATP-dependent RNA helicase DDX56	Oncogene in colorectal cancer and associated with lymphatic invasion and metastasis [102]
CDO1	Cysteine dioxygenase type 1	In CD4 + CTCL, CDO1 overexpression contributed to apoptosis-inhibition and chemoprotection [103]
RPL7L1	60S ribosomal protein L7-like 1	Elusive

* UNG was quantified by SILAC in Jurkat only, but nuclear isoform UNG2 found to be robustly downregulated in SUDHL5 by western analysis, targeted MS and IHC

Ingenuity Pathway Analysis^®^ (IPA) reported that the most confidently affected biological pathway in Jurkat was phagosome maturation (p = 1.69E−6), in agreement with a previous SILAC study in Jurkat [9]. In SUDHL5, the most affected pathway was pyrimidine deoxyribonucleotides de novo biosynthesis I (p = 2.24E−06) (Fig. 1d).

Table 2 shows the ten most up- and downregulated proteins in each of the two cell lines, of which none were quantified in the previous study [9]. Most upregulated in Jurkat was RWDD2B (11.3-fold). No functions have yet been assigned to this protein, but two-hybrid assays have reported physical interactions with the DNA damage-inducible proteins BRCA1 [21] and GADD45G [22, 23]. Apolipoproteins B (APOB) and C (APOC) were 9.7-fold and 6.9-fold upregulated, respectively. APOB constitutes much of the LDL particles that is an important source of cholesterol and lipids in lymphoma cells [24], but a functional role of their differential expression in HDACi-treated lymphoma remains elusive. RETSAT (3.3-fold upregulated) is also involved in lipid metabolism by catalyzing saturation of all-*trans* retinol to form all-*trans*-13,14-dihydroretinol [25]. High levels may drain the all-trans retinol pool and hinder formation of all-*trans* retinoic acid (atRA). Since at RA regulates differentiation, proliferation and apoptosis, inhibition of RETSAT is suggested a novel means to control neoplasms [26].

**Table 2:** 20 most differentially expressed proteins in Jurkat and SUDHL5 after SAHA treatment

Gene symbol	Protein name	Fold change*
*Jurkat up*		
RWDD2B	RWD domain-containing protein 2B	11.3
APOB	Apolipoprotein B-100	9.8
APOC3	Apolipoprotein C-III	6.9
RETSAT	All-trans-retinol 13,14-reductase	3.3
FERMT2	Fermitin family homolog 2	2.8
TUBB2A	Tubulin beta-2A chain	2.6
MT-CO1	Cytochrome c oxidase subunit 1	2.5
TUBB3	Tubulin beta-3 chain	2.3
BAG3	BAG family molecular chaperone regulator 3	2.2
H1FX	Histone H1x	2.1
*Jurkat down*		
UNG	Uracil-DNA glycosylase	− 3.3
TTC4	Tetratricopeptide repeat protein 4	− 2.6
TIMM8B	Mitochondrial import inner membrane translocase subunit Tim8 B	− 2.5
NAF1	H/ACA ribonucleoprotein complex non-core subunit NAF1	− 2.1
ZFAND5	AN1-type zinc finger protein 5	− 2.0
CD3E	T-cell surface glycoprotein CD3 epsilon chain	− 1.9
CD247	T-cell surface glycoprotein CD3 zeta chain	− 1.9
TIMM13	Mitochondrial import inner membrane translocase subunit Tim13	− 1.9
CCDC86	Coiled-coil domain-containing protein 86	− 1.9
CD3D	T-cell surface glycoprotein CD3 delta chain	− 1.9
*SUDHL5 up*		
PIK3CB	Phosphatidylinositol 4,5-bisphosphate 3-kinase cat. subunit beta	8.2
PDCD4	Programmed cell death protein 4	4.5
MGST3	Microsomal glutathione S-transferase 3	3.4
VCL	Vinculin	3.1
HSDL2	Hydroxysteroid dehydrogenase-like protein 2	2.5
PNKD	Probable hydrolase PNKD	2.3
ZNF593	Zinc finger protein 593	2.1
ACO1	Cytoplasmic aconitate hydratase	2.0
H1FX	Histone H1x	2.0
ITPR1	Inositol 1,4,5-trisphosphate receptor type 1	2.0
*SUDHL5 down*		
TYMS	Thymidylate synthase	− 2.9
UBE2C	Ubiquitin-conjugating enzyme E2 C	− 2.7
RRM1	Ribonucleoside-diphosphate reductase large subunit	− 2.6
RRM2	Ribonucleoside-diphosphate reductase subunit M2	− 2.2
SYF2	Pre-mRNA-splicing factor SYF2	− 2.1
CKS2	Cyclin-dependent kinases regulatory subunit 2	− 2.1
LARP4	La-related protein 4	− 2.1
LIMD2	LIM domain-containing protein 2	− 2.0
MPV17L2	Mpv17-like protein 2	− 2.0
RSL24D1	Probable ribosome biogenesis protein RLP24	− 2.0

*Linear fold change

The most upregulated protein in SUDHL5 was the PI3-kinase catalytic subunit PIK3CB isoform (8.2-fold). Uncontrolled PI3K activity mediates defective functions of both B- and T-cells, including reduced CSR and somatic hypermutation (SHM). PDCD4 (programmed cell death protein 4) (4.5-fold upregulated) may reduce cell growth and induce apoptosis by inhibiting the translation initiation factor EIF4A [27]. PDCD4 is a major target of miR-21, an oncomiR in pre-B-cell lymphoma that post-transcriptionally downregulates PDCD4 in a variety of cancers. Since HDAC1 activates miR-21 transcription [28] and is also inhibited by SAHA, this may explain the marked upregulation of PDCD4 in SUDHL5 and contribute to the cytostatic effects of SAHA in CTCL. Inositol 1,4,5-trisphosphate receptor type 1 (ITPR1) was found to be twofold upregulated in SUDHL5. This intracellular receptor plays an important role in the endoplasmic reticulum (ER) stress response, interacts with BCL2 and cytochrome c, and sensitizes cells to apoptotic stimuli [29, 30]. Increased expression of ITPR1 could contribute to the efficacy of the pan-HDACi panobinostat in combination with bortezomib and dexamethasone in the treatment of relapsed or refractory multiple myeloma, which is highly sensitive to ER stress because of extensive immunoglobulin synthesis [31].

The most downregulated protein in Jurkat was UNG (3.3-fold). UNG is the major DNA glycosylase for removal of genomic uracil and exists as mitochondrial (UNG1) and nuclear (UNG2) isoforms encoded by the same gene [32]. Lack of UNG mediates B-cell lymphoma in mice [33] and compromised immunoglobulin (Ig) CSR in humans due to impaired UNG2-mediated excision of uracils induced by activation induced deaminase (AID) at *Ig* switch regions [34]. UNG2 loss may also aggravate genomic instability in APOBEC3B expressing cancers [10]. Notably, three of the four proteins that helps activate the T-cell receptor (TCR) in the TCR complex CD3E (CD3ε), CD247 (CD3ζ) and CD3D (CD3δ) were among the ten most downregulated (1.9-fold) in Jurkat following SAHA treatment (Table 2), in agreement with earlier findings [35] and may explain the mono-therapy effects of HDACis in peripheral T-cell lymphomas such as CTCL, where T cell receptor activation can be a sustaining factor [36].

**Fig. 2:**
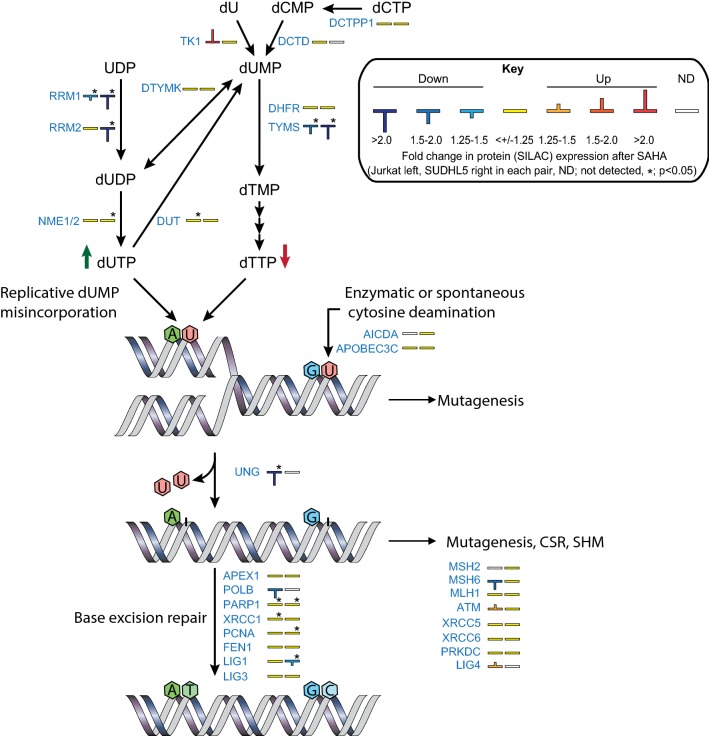
HDAC inhibition mediates downregulation of proteins involved in genomic uracil homeostasis. Enzymes involved in regulation of the cellular dUTP/dTTP ratio as well as processing of genomic uracil are shown, together with their differential expression levels in Jurkat (left bar codes) and SUDHL5 (right bar codes) after SAHA treatment (*; t-test p ≤ 0.05)

In SUDHL5, three key enzymes in the pyrimidine nucleotide biosynthesis pathway were among the most downregulated proteins. Thymidylate synthase (TYMS) was 2.9-fold downregulated, whereas two subunits of ribonucleoside diphosphate reductase (RRM1 and 2) were downregulated 2.6- and 2.2-fold, respectively. TYMS as well as RRM1 were also significantly downregulated in Jurkat (1.9- and 1.4-fold, respectively) (Table 1). Thus, it seems that levels of key enzymes governing the cellular dUTP/dTTP ratio are preferentially affected by HDACi in both the T- and B- cell lines (Fig. 2). The SILAC data also revealed marked (2.3–11-fold) downregulation of the PCNA-associated factor PCLAF (PAF15) in SUDHL5 but was reported non-significant due to the large variation. PCLAF was also found to be downregulated, although weaker (1.1–1.9-fold) in Jurkat. PCLAF is an oncogenic protein and a central regulator of DNA translesion synthesis (TLS) by promoting binding of DNA polymerase δ to proliferating cell nuclear antigen (PCNA) and restraining recruitment of the TLS DNA polymerase η. Since PCLAF binds to the same interaction motif in PCNA as p21^Cip1^ (CDKN1A) and p33^Ing1^ (ING1), the PIP-box, high levels may overcome p21-mediated cell cycle arrest and p33-mediated cell death (reviewed in [37]). Interestingly, binding of PCLAF to PCNA is mediated by monoubiquitinylations of PCLAF Lys^15^ and Lys^24^, recently demonstrated to be catalyzed by UHRF1 [38]. UHRF1 is one of the proteins commonly downregulated in Jurkat and SUDHL1 after SAHA treatment (Fig. 1c). Combined depletion of UHRF1 and PCLAF might thus co-operate to the anti-tumorigenic effects of HDACi in lymphoma treatment. It should be noted that we also observed downregulation of PCLAF in the non-lymphoma human cell lines, HEK293 (embryonal kidney), HeLa (cervical carcinoma), DLD1 (colon carcinoma) and HaCaT (skin keratinocytes) (Additional file 2: Figure S1), indicating that this might a common effect of HDACi-treatment in several cell types, including cancer cells. Finally, we found a significant downregulation BCL6 (1.5-fold, p = 0.001) in SUDHL5 (Additional file 1: Table S1), in agreement with a very recent study demonstrating romidepsin-mediated depletion of BCL6 in B-cell lymphoma cells [39]. BCL6 is a central mediator of germinal center formation [40] and has been suggested as a therapeutic target in lymphoma [41].

### HDAC1/3 inhibition induces robust depletion of nuclear UNG2 in several cell lines

**Fig. 3:**
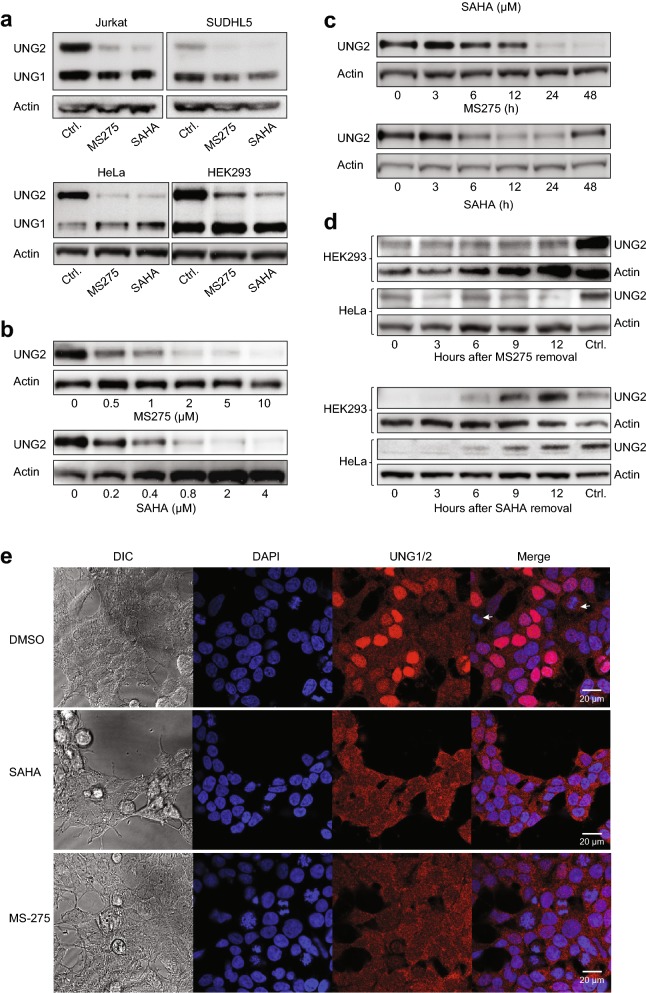
HDAC inhibition mediates selective depletion of the nuclear UNG2 isoform in various cell lines. **a** Western analysis of TCEs from Jurkat, SUDHL5, HeLa and HEK293 cells using a polyclonal antibody (PU059) recognising the common catalytic domain of nuclear UNG2 and mitochondrial UNG1. The membranes were subsequently probed with anti-actin antibodies as loading controls. Robust depletion by both 5 µM MS-275 and 2 µM SAHA was specific for the UNG2 isoform. **b** HEK293 cells were treated with various concentrations of SAHA or MS-275 for 24 h, and TCEs were subject to western analysis as in **a**. **c** HEK293 cells were treated with 2 µM SAHA or 5 µM SAHA, harvested at different time points and subject to western analysis as in **a**. **d** Induction of UNG2 expression after HDACi removal. After 18 h culture of HEK293 and HeLa cells in media containing 5 µM MS-275 (top panels) or 2 µM SAHA (bottom panels) with optimal depletion, cells were cultured in media without HDACi and UNG2 expression monitored by western analysis at the given time points. **e** DAPI nuclear staining (blue) and immunocytochemical staining of UNG1/2 (red) in HEK293 cells treated with 2 µM SAHA, 5 µM MS-275 or DMSO vehicle for 24 h prior to immunostaining with polyclonal PU59 antibody. Note the selective depletion of nuclear UNG2 whereas mitochondrial UNG1 remains unaffected after HDACi treatment. The differential UNG2 staining in the DMSO controls reflects the strict cell-cycle dependent expression of the nuclear UNG2 isoform, which peaks in late G1/S and is lowest in G2 and M-phase (white arrows indicate mitotic cells). DIC; Differential interference contrast images of the same sections

Large-scale analysis of mutational signatures across human cancers strongly suggest that dysregulated deamination of DNA cytosine to uracil by the apolipoprotein B mRNA editing enzyme, catalytic polypeptide-like (APOBEC)/AID family of deaminases plays an important role in tumorigenesis [42, 43]. The repair of genomic uracil, originating from such deamination or by misincorporation during DNA synthesis, is mainly performed by UNG1 in the mitochondria and UNG2 in the nucleus. To examine to what extent the two isoforms, that differ only in the N-terminal sequence and hence size, were individually affected by HDACi, Jurkat and SUDHL5 as well as HeLa and HEK293 cells were treated with SAHA or MS-275, and UNG1/2 isoforms quantified by western analysis of the protein extracts. Strikingly, in all four cell lines both HDACi induced robust depletion of the nuclear 36.5 kDa UNG2, while the 31 kDa mature mitochondrial UNG1 was much less affected (Fig. 3a). UNG2 depletion was dose-dependent with maximal reduction at the initially tested concentrations of 2 µM and 5 µM for SAHA and MS-275, respectively (HEK293 in Fig. 3b, HeLa not shown). UNG2 levels started decreasing between 6 and 12 h after treatment, and very low amounts remained after 24 h with both inhibitors (Fig. 3c). At 48 h of MS-275 treatment, UNG2 was barely detectable, whereas UNG2 re-expression was observed at this time point after SAHA treatment. This is likely explained by the shorter in vivo half-life of SAHA compared to MS-275 [44, 45]. The differential mode of action of MS-275 and SAHA was further underscored by replacing the HDACi-containing cell culture medium with fresh medium after 24 h HDACi treatment. Here, re-expression of UNG2 was evident at 6 h after SAHA removal in both cell lines, whereas robust depletion was still observed at 12 h after MS-275 removal (Fig. 3d). Cells were also treated with the pan-HDACi valproate or sodium butyrate, both of which depleted UNG2 (data not shown). UNG2 degradation was further replicated with HDACi treatment in DLD1 and HaCaT, as well as in A549 (human lung carcinoma), and HAP1 (human near-haploid chronic myelogenous leukemia) cells. (Additional file 3: Figure S2). Finally, immunoconfocal microscopy showed UNG staining in both cytoplasm (UNG1) and nuclei (UNG2) of untreated HEK293, whereas SAHA- or MS-275-treated cells displayed markedly reduced nuclear staining (Fig. 3e) in agreement with western and SILAC analyses. The latter was also observed in HeLa cells treated with SAHA or MS-275 (Additional file 4: Figure S3). In summary, these results suggest that HDACi mediate selective and ubiquitous depletion of the UNG2 isoform in human cells.

### HDACi-induced depletion of UNG2 is independent of altered transcription or cell cycle status

**Fig. 4:**
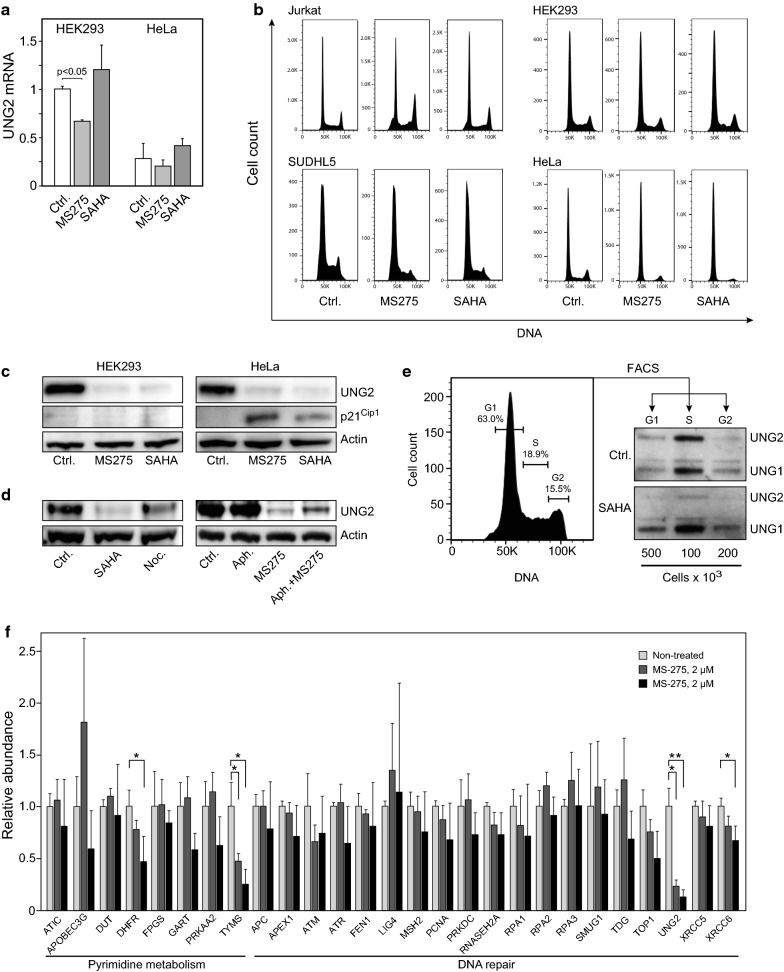
**a** Quantitative RT-PCR of UNG2 mRNA isolated from HeLa and HEK293 cells treated with 2 µM SAHA, 5 µM MS-275 or DMSO for 24 h. **b** Flow cytometry histograms of HEK293-, HeLa-, Jurkat- and SUDHL5 cells treated with 2 µM SAHA, 5 µM MS-275 or DMSO for 24 h. **c** Expression of p21^Cip1^ in HEK293- (left) and HeLa cells (right) treated as in **c**. **d** Treatment of HEK293 with cells 2 µM SAHA for 24 h mediated a stronger depletion of UNG2 than the G2/M-blocking agent nocodazole (10 µM, 24 h) (left panels). MS-275 mediated robust inhibition of UNG2 in cells arrested in G1/S by co-treatment with aphidicolin (10 µM, 24 h) (right panels). **e** HEK293 cells were treated with 2 µM SAHA for 12 h and subjected to fluorescence-activated cell sorting into G1-, S- and G2 fractions. Percentages of cells in each cell cycle phase are indicated in the flow cytometry histogram (left panel) and western blots showing UNG2 expression in SAHA- and DMSO -treated cells (right panels). **f** Expression of 27 selected proteins involved in pyrimidine metabolism as well as DNA repair were quantified by PRM. Each bar represents the mean of at least three biological replicates with SDs as indicated (_*_ p < 0.05). Proteotypic peptides employed in the PRM analyses are given in Additional file 6: Table S2

In freely cycling cells UNG2 expression peaks in late G/early S and diminishes in G2/M due to reduced mRNA expression [46] and increased proteasomal degradation [47]. To measure contribution of altered transcript levels, we quantified UNG2 mRNA in HeLa and HEK293 treated with MS-275 or SAHA for 24 h. Whereas no significant change was observed after SAHA-treatment, a modest (30%) reduction in UNG2 mRNA was observed in MS-275-treated HEK293 cells (Fig. 4a). The magnitude of this reduction could not, however, explain the robust UNG2 depletion observed at the protein level. Further, to investigate whether HDACi-induced cell cycle arrest in G2/M could contribute to UNG2 depletion in the different cell lines by increasing canonical UNG2 turnover, Jurkat, SUDHL5, HEK293 and HeLa cells were subjected to flow cytometric analysis after MS-275 or SAHA treatment (Fig. 4b). HDACi treatment mediated a modestly increased G2 fraction in Jurkat, while G2 was reduced in HeLa and SUDHL5 and minimally affected in HEK293. Notably, HeLa also displayed a markedly reduced S-phase fraction, potentially explained by induction of p21^Cip1^, which was not observed in HEK293 cells (Fig. 4c). Thus, accumulation of cells in G2 was evidently not the underlying cause of HDACi-mediated UNG2 depletion. This was further supported by treatment of HEK293 cells with either the G2/M-blocker nocodazole or the G1/S-blocker aphidicolin (Fig. 4d). Here, SAHA mediated even stronger UNG2 depletion than nocodazole. Furthermore, MS-275 was able to mediate strong depletion of UNG2 even in cells synchronized in G1/S by aphidicolin treatment. The ability of HDAC inhibition to deplete UNG2 in all cell cycle phases was finally demonstrated by fluorescence-activated cell sorting (FACS^®^) of HEK293 G1-, S- and G2 fractions. As shown in Fig. 4e, SAHA mediated prominent UNG2 depletion (relative to UNG1) in all three cell cycle phases and virtually complete loss in G1 and G2 fractions.

Many DNA repair proteins as well as proteins involved in deoxynucleotide metabolism are known to be cell-cycle regulated [48, 49]. We thus undertook an in-depth study of selected proteins in HEK293 cells where the cell cycle was least affected by the treatment (Fig. 4b). In addition to the above dose of 5 µM MS-275, we employed a reduced dose of 2 µM, at which cell viability was essentially unaffected (Additional file 5: Figure S4). Three biological replicates at each concentration as were subjected to targeted quantitative analysis by parallel reaction monitoring (PRM) encompassing 27 selected proteins, including additional targets not quantified in the SILAC data (proteotypic PRM peptides are given in Additional file 6: Table S2). Only UNG2 and TYMS were significantly depleted by 2 µM MS-275. At 5 µM MS-275, UNG and TYMS were further reduced, and additional significant depletion of DHFR and XRCC6 (Ku70) was observed (Fig. 4f). These results underscore that HDAC inhibition markedly deplete levels of enzymes involved in regulating cellular dUTP/dTTP levels, supporting earlier studies reporting HDACi-mediated TYMS and DHFR depletion [50, 51]. Moreover, among the DNA repair proteins, UNG2 appear to be specifically targeted by class 1/3 HDACi.

### Degradation of UNG2 by HDACi occurs by increased proteasomal degradation, accompanied by novel acetylated isoforms

**Fig. 5:**
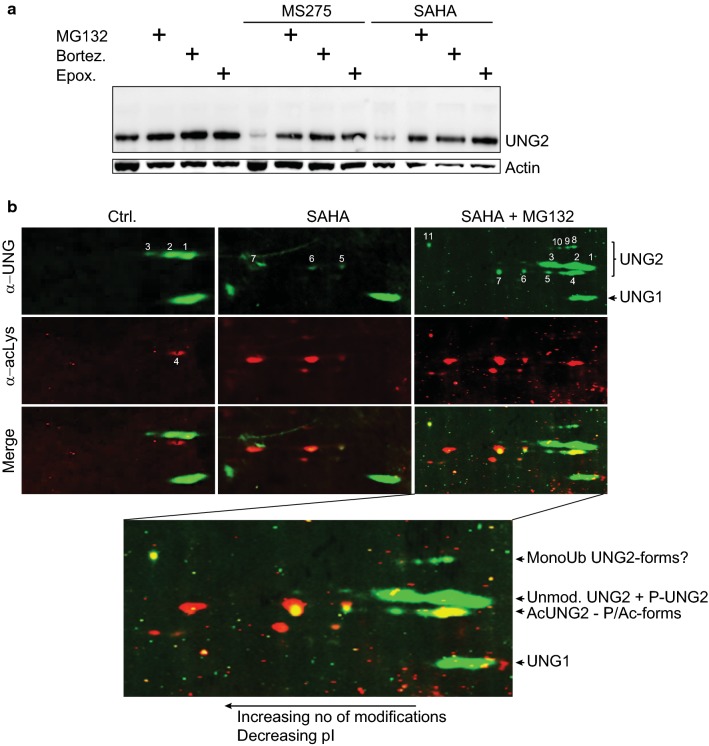
**a** Proteasome inhibitors prevent HDACi-mediated UNG2 depletion. HEK293 cells were treated for 24 h with 5 µM MS-275, 2 µM SAHA or DMSO control in the presence or absence of proteasome inhibitors as indicated (10 µM MG132, 1 µM epoxomicin, 10 µM bortezomib). **b** 2D-PAGE blots of HEK293 cell extracts after 24 h treatment with DMSO, 2 µM SAHA or 2 µM SAHA + 10 µM MG132 probed with anti-UNG2 PU59 polyclonal antibodies and anti acetyllysine monoclonal antibodies, followed by Alexa fluor 800 (anti-rabbit) or 680 (anti-mouse) secondary antibodies, respectively. Control cells (left panels), showed a pattern of UNG2 isoforms resembling that earlier observed [47], consisting of unmodified, mono- and diphosphorylated UNG2 (designated 1, 2 and 3, respectively). A slightly faster migrating acetylprotein, designated 4 could represent an acetylated isoform as acetylation may induce faster electrophoretic migration and acidic shift in 2D-PAGE, compared to their unmodified forms [84, 85]. The middle panel show cells treated with SAHA (2 µM, 24 h). Strikingly, UNG2 forms 1, 2 and 3 are absent, instead, three novel and faint UNG2 isoforms (designated 5, 6 and 7) are evident, overlapping with acetyllysine signals. Combined SAHA (2 µM) treatment and proteasome inhibition by MG132 (10 µM) (right panels) restored isoforms 1, 2 and 3 and enhanced the acetylated forms 5, 6 and 7. Strikingly, strong UNG2 and acetyl staining was now observed conforming to isoform 4 in the control cells. Several other faint UNG2-positive spots were observed, with varying degree of acetylation signal (bottom enlarged panel). In addition, several isoforms having electrophoretic 2nd dimension migration conforming to monoubiquitinylated UNG2 [47] were observed, of which isoforms 8, 9 and 10 overlapped with weak-, and isoform 11 with strong acetyllysine staining. Notably, isoform 11 conforms well to a multiple phosphorylated and mono-ubiquitinylated isoform of UNG2 observed in G2-phase HeLa [47]. Here we also observe faint signal in S-phase corresponding to isoforms 4, 5, 6, and may indicate a role of acetylation of UNG2 in S-phase (Fig. 4e)

To identify the mechanism underlying UNG2 depletion in more detail, HEK293 cells were treated with MS-275 or SAHA in the presence of various protease inhibitors. Among these, the autophagosome-inhibitor bafilomycin A1 and cell-permeable calpastatin peptide, inhibiting calpain I/II, did not alter UNG2 levels (data not shown). Conversely, each of the proteasomal inhibitors MG132, bortezomib and epoxomicin elevated endogenous levels in monotherapy and mediated near full recovery of UNG2 expression without inducing bands representing potentially ubiquitinylated forms of UNG2 (Fig. 5a).

UNG2 is extensively regulated by PTMs, likely to fine-tune its activity in different genomic contexts as well as through the cell cycle. We have previously identified phosphorylated isoforms as well as mono-ubiquitination [47], whereas several acetylated lysines have been reported in large-scale acetylome studies [52]. Most of the reported UNG acetyllysines reside in the common catalytic domain and could thus not be assigned to either UNG1 or UNG2 isoforms by mass spectrometry analysis alone. To analyze potential alteration of UNG2 PTM-status subsequent to HDACi, cell lysates of HEK293 treated with SAHA or MG132 were separated by two-dimensional (2D)-PAGE before electroblotting and probing for acetyllysine and UNG with different secondary IRDye 800/680 antibodies, allowing precise overlap of differentially acetylated isoforms. The highly complex pattern of UNG2 isoforms rendered it difficult to delineate the stepwise modifications and we were not able to determine the lysine residues by mass spectrometry due to the low stoichiometry (Fig. 5b and legend). In conclusion, however, HDACi treatment alone mediates hyperacetylation of UNG2, and combined HDACi and proteasomal inhibition mediates accumulation of unmodified and acetylated isoforms as well as minor additional isoforms that are apparently mono-ubiquitinylated, supporting a role of acetylation in proteasomal degradation of UNG2.

### Murine CH12F3 B-cells show reduced class-switch recombination after HDACi treatment

**Fig. 6:**
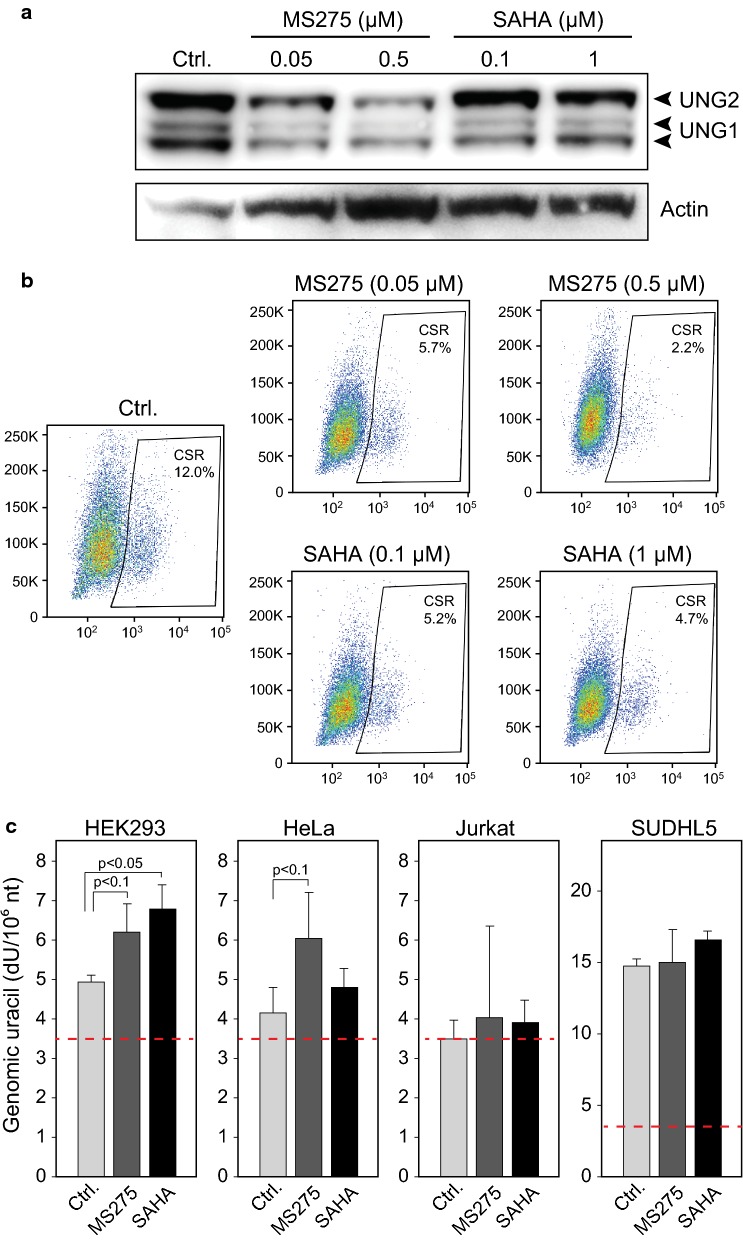
**a** Murine CH12F3 cells were subjected to HDACi treatment at the given concentrations for 48 h prior to western analysis of UNG1/2 expression. **b** Representative flow cytograms illustrating dose-dependent reduction in IgM to IgA class switching in CH12F3 cells with induction treatment and addition of HDACi as indicated. **c** Quantification of genomic uracil in HEK293, HeLa, Jurkat and SUDHL5 cells after 48 h treatment with 2 µM SAHA or 5 µM MS-275 for 48 h. Red dashed line indicates dU level in UNG-treated DNA control (detection limit)

HDACi have gained growing attention for their capability to modulate B-cell mediated immune-responses, in which genomic uracil induction by AID and excision by UNG2 are central intermediates. In human and mouse B-cells, HDACi inhibit CSR, SHM and plasma cell differentiation, and this was attributed to reduced protein levels of AID and PRDM1 (BLIMP-1) [53], the latter of which drives maturation of plasma cells. The same study also monitored expression of several other factors involved in antibody affinity maturation, but at their mRNA levels only. HDACi mediated significant reduction in Xbp1 mRNA, which gene product promotes Ig secretion [54], whereas Ung mRNA remained unaffected, in agreement with our findings. To further investigate a potential contribution of Ung depletion to the HDACi-mediated dampening of CSR, we treated murine CH12F3 cells with two concentrations of either SAHA or MS-275, of which the lowest dose did not mediate any significant effect upon viability (Additional file 7: Figure S5). Murine Ung2 was depleted subsequent to treatment with either HDACi, although most pronounced with MS-275 (Fig. 6a). Surprisingly, the treatment also mediated significant depletion of Ung1, which is highly relevant since we recently demonstrated that a murine Ung1 isoform is targeted to the nucleus and can support CSR in CH12F3 cells [20]. Concomitant with the Ung1/2 reduction, MS-275 mediated a dose-dependent decrease in CSR and a > fivefold reduction at the 0.5 µM concentration (Fig. 6b). CSR was also inhibited at both concentrations of SAHA, although to a lesser extent than with MS-275, in agreement with the western results. The quantitative contribution of AID- versus UNG depletion to the HDACi-mediated CSR inhibition could not be determined from our experiments. Nevertheless, a large body of evidence, including work from our own laboratory, has demonstrated the critical function of UNG in CSR [34, 55–57], including formation of class switched autoantibodies mediating autoimmune disease [58].

### HDACi mediate increased genomic uracil in freely cycling HEK293 cells

To evaluate whether HDACi-mediated depletion of UNG2 and TYMS resulted in detectable alteration in genomic uracil or other nucleobase modifications, HEK293, HeLa, Jurkat and SUDHL5 were treated with either SAHA or MS-275 prior to LC–MS/MS quantification of non-canonical nucleobases. Fetal bovine serum (FBS) may contain thymidine [59] and was thus dialyzed prior to addition the culture media to reduce salvage dTTP synthesis. Since UNG2 has been implicated in TET-mediated DNA demethylation [60], we also quantified genomic 5-methylcytosine (5mC), 5-hydroxymethylcytosine (5hmC) and 5-formylcytosine (5fC). Whereas no changes were observed in the genomic levels of 5-meC or the demethylation intermediates 5-hmC and 5-fC (data not shown), HDACi treatment apparently mediated increased genomic uracil in all four cell lines. However, this increase was only statistically significant in HEK293 after SAHA treatment (p < 0.05) and not significant (p < 0.1) in HEK293 and HeLa after MS-275 treatment (Fig. 6c). It should be noted that the steps involved in quantification of genomic uracil inevitably produce cytosine deamination and background uracil levels as indicated by red dashed lines in Fig. 6c, and which negatively affects statistical power. A potential explanation to the differential impact on the genomic uracil levels, is the varying effects of the HDACi upon the cell cycle in the four cell lines (Fig. 4c). Since misincorporation is likely by far the dominating source of genomic uracil in replicating cells ([61] and references therein) the highest rate of misincorporation would be expected in HEK293. The more pronounced increase in genomic uracil by SAHA than MS-275 conforms to the stronger depletion of UNG2 by SAHA in this cell line (Fig. 3a). It should be noted that depletion of UNG2 alone in not likely to contribute to the cytotoxic effects of HDACi. Human cells contain three other uracil-DNA glycosylases (SMUG1, TDG and MBD4) that exert backup functions in various genomic contexts ([62] and references therein) and this redundancy likely contribute to the fact that homozygous UNG-deficiency is not lethal in humans or mice. Nevertheless, the observed HDACi-mediated increase in genomic uracil might contribute to genomic instability as well to transcriptional alterations in the treated cells, since replacement of canonical DNA bases in certain transcription-factor binding sites can profoundly affect transcription efficiency [63].

## Discussion

SILAC-based proteome profiling in SAHA-treated T- and B-lymphoma cell lines revealed 19 common DEPs in Jurkat and SUDHL5. Such commonly affected proteins may be of significant clinical interest since they potentially mirror ubiquitous effects of HDACi treatment. Several of the common DEPs as well as and the most up-or downregulated proteins in either cell line are highly relevant to cellular proliferation or survival, as well as to responses to chemotherapeutics (Table 1 and references therein). Previous studies have demonstrated that class I/II HDACi deregulate DNA double strand break repair by homologous recombination and non-homologous end-joining (NHEJ) [7] and may attenuate nucleotide excision repair (NER) [64]. The robust and consistent reduction of UNG2 observed in the present study establishes DNA base-excision repair (BER) among the DNA repair pathways affected by this class of inhibitors and substantiates that deregulated DNA repair might be an indirect cause of genotoxic effects observed with some HDACi [65]. Importantly, this does not preclude the use of HDACi in cancer treatment but should be taken into consideration during their risk assessment. Moreover, HDACi-mediated inhibition of certain DNA repair pathways can be exploited to increase efficacy and/or to overcome drug resistance. Increased cytotoxicity of 5-fluorouracil (5-FU) has been reported when combined with HDACi [66, 67] and one study has demonstrated that loss of UNG re-sensitizes cells to 5-fluorodeoxyuridine (5-FdU) [68]. Our findings provide a mechanistic explanation for these observations and warrant further investigation on combinatorial effects of HDACi- and antifolate regimens. Our findings also warrant studies of HDACis in melanoma and liver cancer, in which high UNG mRNA expression is an unfavorable prognostic marker (http://proteinatlas.org). The observed depletion of PCLAF suggests that TLS might be an additional novel target of HDACi. Like DNA repair, a considerable body of evidence indicates that TLS contributes chemo- and radioresistance in cancer, and inhibition or depletion of specific TLS factors can overcome this resistance [69, 70]. Currently, no chemical inhibitors of PCLAF are available. However, shRNA-mediated knockdown of PCLAF inhibits TLS and reduces resistance to radiotherapy in glioblastoma multiforme, especially within the glioma stem cell pool [71]. This warrants further studies on HDACi combinatorial treatments in cancers harboring enhanced TLS.

This study underscores the value of proteomic- in addition to transcriptomic analyses to evaluate biological responses to HDACi treatment. Although altered transcriptional regulation due to chromatin remodeling has been proposed to constitute the major mechanism whereby HDACi exert their function [72], we speculate whether hyperacetylation of non-histones is underestimated as a modulator. Among the 40 proteins listed in Table 1, 75% have been reported as carrying acetyllysine modifications, a marked over-representation compared to the overall human proteome (30%) (protein lysine modification database, PLMD 3.0, http://plmd.biocuckoo.org). In addition, 50 out of the 95 acetyllysine residues reported in the proteins from Table 1, are dual acetyl/ubiquitin targets, which might as well regulate protein lifetime.

Here we demonstrate that HDACi-induced UNG2 depletion is mediated by polyubiquitin-independent proteasomal degradation accompanied by hyperacetylation. The exact steps involved in the degradation process remain to be determined. In one possible scenario, specific acetylations directly recruits UNG2 to proteasomal degradation. Within the DNA damage response, ubiquitin-independent degradation is observed among hyperacetylated histones subsequent to replication stress and is facilitated by the PA200-20S proteasome [73, 74]. Such a mechanism might also involve PTM-mediated release of UNG2 from stabilizing protein complexes. The largely unstructured N-terminal region of UNG2 [75] harbors binding motifs to both PCNA and replication protein A (RPA) [47, 76]. We have previously demonstrated two phosphorylations close to the RPA binding motif (pT60 and pS64) that occur in late S-phase and reduce binding to RPA [47], just prior to the decline of UNG2 that results in diminished levels in G2/M. Interestingly, one out of the three acetyllysines reported in large-scale acetylome studies [46, 71] resides in the PCNA-binding motif (K5Ac), whereas the second (K49Ac) resides about 20 residues N-terminal to the RPA-binding motif. Notably, both lysines have also been reported as targets for ubiquitinylation [77, 78]. To what degree their acetylation mediate release from PCNA/RPA and promote proteasomal degradation as well as the potentially concomitant contribution of phosphorylation and mono-ubiquitinylation, is a topic of further study in our laboratory. This could potentially shed light on mechanisms directing UNG2 to uracil processing in various genomic contexts, including replicating/non-replicating chromatin, centromeres and *Ig* loci.

HDACis are known to inhibit CSR, SHM and plasma cell differentiation and this has primarily been attributed to reduced AID and PRDM1 expression [79, 80]. Whereas depletion of AID abrogates cytosine deamination at *Ig* loci, UNG2 has an important role in excision of AID-generated uracils and promote mutagenic processing of the resultant apyrimidinic (AP)-sites [34]. Thus, depletion of UNG2 may contribute to the observed effects of HDACi on both immune- and autoimmune responses. Further, HDACi promote reversal of HIV-1 latency by increasing the LTR activity [5, 81], a major obstacle to permanently eradicate infection [82]. Loss of UNG also increase LTR activity [83] and the potential contribution of HDACi-mediated UNG2 depletion in reversal of HIV-1 latency warrants further investigation. Additional proteomic analyses within these areas must include analysis of freshly prepared lymphocytes. Since freshly isolated human lymphocytes are not easily amenable to metabolic labelling, alternative quantitative mass spectrometry methods including label-free quantification or targeted methods should be employed. For targeted analysis, candidate proteins reported in the present study should provide a valuable source for the synthesis of proteospecific peptides.

HDACi-mediated depletion of UNG2 could also be a means to improve the efficiency of gene therapy. The fusion of APOBEC1 deaminase with CRISPR/dCas9 is a promising method of delivering targeted point mutations for treatment for genetic diseases by introducing site-directed deamination of C to U [11]. To avoid excision of the uracil and faithful repair of the lesion back to cytosine, the bacteriophage inhibitor protein Ugi was fused to dCas9-APOBEC1 to inhibit UNG2. The addition of Ugi increased the editing frequency about threefold. Conceivably, HDACi-treatment prior to- or during base editing could increase editing frequency by e.g. altering chromatin density and further reduce cellular UNG2 activity.

Finally, depletion of UNG was recently shown to induce synthetic lethality in APOBEC3B overexpressing cancers [10]. Many cancers overexpress APOBEC3B or harbor high kataegis mutational signatures conforming to deamination by the APOBEC/AID family of cytidine deaminases. In such cases, HDACi-mediated UNG2 depletion might potentially induce synthetic lethality and thus provide novel treatment options for a large subgroup of cancers.

## Conclusion

The steady-state levels of proteins depend on both translational speed and protein lifetime, neither of which can be inferred by transcriptome studies alone. Thus, our study provides better understanding of the molecular pathways affected by HDACi treatment of B- and T-cell lymphoma cell lines. The apparently ubiquitous dysregulation of proteins affecting genomic uracil induction and processing warrants further investigation to exploit HDACi in combinatorial treatments of cancer, autoimmune disease and HIV-1.

## Supplementary information

**Additional file 1: Table S1.** SILAC quantification data.

**Additional file 2: Figure S1.** HDACi mediate downregulation of PCLAF (PAF15).

**Additional file 3: Figure S2.** HDACi mediate selective depletion of the UNG2 isoform in cell lines of different origin.

**Additional file 4: Figure S3.** Immunoconfocal analysis of UNG1/2 in HDACi-treated HeLa cells.

**Additional file 5: Figure S4.** Viability of HEK293 cells at various concentrations of MS-275.

**Additional file 6: Table S2.** Proteotypic PRM peptides.

**Additional file 7: Figure S5.** Viability of CH12F3 cells at various concentrations of MS-275.

## Data Availability

The SILAC mass spectrometry proteomics data have been deposited to the ProteomeXchange Consortium (16) via the PRIDE partner repository with the dataset identifier PXD00008293. Username: reviewer80971@ebi.ac.uk, Password: rOrcSPjg.

## Abbreviations

AID: Activation induced deaminase
APOBEC: Apolipoprotein B mRNA editing enzyme, catalytic polypeptide-like
BER: Base excision repair
CSR: Class switch recombination
DEP: Differentially expressed protein
FACS^®^: Fluorescence-activated cell sorting
HAT: Histone acetyltransferase
HDAC: Histone deacetylase
HDACi: Histone deacetylase inhibitor
UNG1/2: Uracil-DNA glycosylase isoform 1/2
PCLAF: PCNA clamp associated factor
PCNA: Proliferating cell nuclear antigen
RPA: Replication Protein A
SAHA: Suberanilohydroxamic acid
SHM: Somatic hypermutation
SILAC: Stable isotope labeling with amino acids in cell culture
TCE: Total cell extract
TLS: Translesion synthesis
TYMS: Thymidylate synthase
