# Correction to: HDACi mediate UNG2 depletion, dysregulated genomic uracil and altered expression of oncoproteins and tumor suppressors in B- and T-cell lines

**DOI:** 10.1186/s12967-021-02896-1

**Published:** 2021-06-04

**Authors:** Tobias S. Iveland, Lars Hagen, Animesh Sharma, Mirta M. L. Sousa, Antonio Sarno, Kristian Lied Wollen, Nina Beate Liabakk, Geir Slupphaug

**Affiliations:** 1 Department of Clinical and Molecular Medicine, Faculty of Medicine and Health, Norwegian University of Science and Technology, 7491 Trondheim, Norway; 2 Cancer Clinic, St. Olav’s Hospital, Trondheim, Norway; 3 Clinic of Laboratory Medicine, St. Olav’s Hospital, Trondheim, Norway; 4 Proteomics and Modomics Experimental Core, PROMEC, at NTNU and the Central Norway Regional Health Authority, Stjørdal, Norway

## Correction to: J Transl Med (2020) 18:159 https://doi.org/10.1186/s12967-020-02318-8

The original publication of this article [1] contained 2 incorrect PRIDE identifiers. In this correction article the incorrect and correction information is published. The original article has been updated.

**Incorrect**

- The mass spectrometry proteomics data have been deposited to the ProteomeXchange Consortium [16] via the PRIDE partner repository with the dataset identifier **PXD00008293**.

**Correct**

- The mass spectrometry proteomics data have been deposited to the ProteomeXchange Consortium [16] via the PRIDE partner repository with the dataset identifier **PXD008293**.

**Incorrect**

- The SILAC mass spectrometry proteomics data have been deposited to the ProteomeXchange Consortium (16) via the PRIDE partner repository with the dataset identifier **PXD00008293**.

**Correct**

- The SILAC mass spectrometry proteomics data have been deposited to the ProteomeXchange Consortium (16) via the PRIDE partner repository with the dataset identifier **PXD008293**.
