# Supplementary material for: HDACi mediate UNG2 depletion, dysregulated genomic uracil and altered expression of oncoproteins and tumor suppressors in B- and T-cell lines
Source: J Transl Med. 2020 Apr 7;18:159. doi: 10.1186/s12967-020-02318-8 (PMC7137348; doi:10.1186/s12967-020-02318-8)
Supplement: Supplementary file 6 — Additional file 6: Table S2. Proteotypic PRM peptides. [file 12967_2020_2318_MOESM6_ESM.docx]

**Table S2. List of peptides used for quantitative parallel reaction monitoring (PRM)**

| **Gene** | **Protein** | **Peptide** |
| --- | --- | --- |
|  |  |  |
| APC | Adenomatous polyposis coli protein | LNSFIQVDAPDQK |
| APEX1 | DNA-(apurinic or apyrimidinic site) lyase | EAAGEGPALYEDPPDQK |
|  |  | EGYSGVGLLSR |
|  |  | VSYGIGDEEHDQEGR |
| APOBEC3G | DNA dC->dU-editing enzyme APOBEC-3G | LYYFWDPDYQEALR |
| ATIC | Bifunctional purine biosynthesis protein PURH | EALGIPAAASFK |
|  |  | NGQVIGIGAGQQSR |
| ATM | Serine-protein kinase ATM | LQQTAFENAYLK |
| ATR | Serine/threonine-protein kinase ATR | SEWTDELNTYR |
|  |  | APLNETGEVVNEK |
| DUT | Deoxyuridine 5'-triphosphate nucleotidohydrolase | TDIQIALPSGCYGR |
|  |  | GNVGVVLFNFGK |
|  |  | IAQLICER |
|  |  | IFYPEIEEVQALDDTER |
| DHFR | Dihydrofolate reductase | LTEQPELANK |
|  |  | LLPEYPGVLSDVQEEK |
| FEN1 | Flap endonuclease 1 | LIADVAPSAIR |
|  |  | YPVPENWLHK |
|  |  | WSEPNEEELIK |
| FPGS | Folylpolyglutamate synthase | INGQPISPELFTK |
| GART | Trifunctional purine biosynthetic protein adenosine-3 | DPLLASGTDGVGTK |
| LIG4 | DNA ligase 4 | LILPQLER |
|  |  | HLYIGGDDEPQEK |
|  |  | ESWVTDSIDK |
| MSH2 | DNA mismatch repair protein Msh2 | GDFYTAHGEDALLAAR |
|  |  | LYQGINQLPNVIQALEK |
|  |  | TEYEEAQDAIVK |
|  |  | NNSFVNEIISR |
| PCNA | Proliferating cell nuclear antigen | SEGFDTYR |
|  |  | CAGNEDIITLR |
|  |  | AEDNADTLALVFEAPNQEK |
|  |  | DLSHIGDAVVISCAK |
|  |  | FSASGELGNGNIK |
| PRKAA2 | 5'-AMP-activated protein kinase catalytic subunit alpha-2 | IGHYVLGDTLGVGTFGK |
|  |  | SIDDEVVEQR |
| PRKDC | DNA-dependent protein kinase catalytic subunit | VTELALTASDR |
|  |  | DPESETDNDSQEIFK |
|  |  | NELEIPGQYDGR |
| RNASEH2A | Ribonuclease H2 subunit A | LQDLDTDYGSGYPNDPK |
| RPA1 | Replication protein A 70 kDa DNA-binding subunit | MFILSDGEGK |
|  |  | EDSHPFDLGLYNEAVK |
| RPA2 | Replication protein A 32 kDa subunit | QWVDTDDTSSENTVVPPETYVK |
| RPA3 | Replication protein A 14 kDa subunit | FIVNTLK |
|  |  | VVPIASLTPYQSK |
|  |  | FFPLIEVNK |
| SMUG1 | Single-strand selective monofunctional uracil DNA glycosylase | NLTPAELPAK |
|  |  | LNELGLLPLLLK |
| TDG | G/T mismatch-specific thymine DNA glycosylase | FNGVSEAELLTK |
|  |  | NLEFGLQPHK |
| TOP1 | DNA topoisomerase 1 | AEEVATFFAK |
|  |  | TYNASITLQQQLK |
|  |  | ELTAPDENIPAK |
| TYMS | Thymidylate synthase | TGTGTLSVFGMQAR |
|  |  | DFLDSLGFSTR |
|  |  | EEGDLGPVYGFQWR |
|  |  | DMESDYSGQGVDQLQR |
| UNG2 | Uracil-DNA glycosylase, nuclear isoform | TLYSFFSPSPAR |
| XRCC5 | X-ray repair cross-complementing protein 5 | LGGHGPSFPLK |
|  |  | TDTLEDLFPTTK |
| XRCC6 | X-ray repair cross-complementing protein 6 | DSLIFLVDASK |
|  |  | DIISIAEDEDLR |
